# Identification of Spring Wheat with Superior Agronomic Performance under Contrasting Nitrogen Managements Using Linear Phenotypic Selection Indices

**DOI:** 10.3390/plants11141887

**Published:** 2022-07-20

**Authors:** Muhammad Iqbal, Kassa Semagn, J. Jesus Céron-Rojas, José Crossa, Diego Jarquin, Reka Howard, Brian L. Beres, Klaus Strenzke, Izabela Ciechanowska, Dean Spaner

**Affiliations:** 1 Department of Agricultural, Food and Nutritional Science, 4-10 Agriculture-Forestry Centre, University of Alberta, Edmonton, AB T6G 2P5, Canada; mi1@ualberta.ca (M.I.); strenzke@ualberta.ca (K.S.); izabela@ualberta.ca (I.C.); 2 Biometrics and Statistics Unit, International Maize and Wheat Improvement Center (CIMMYT), Km 45 Carretera, Veracruz 52640, Mexico; jesusceronrojas@live.com.mx (J.J.C.-R.); j.crossa@cgiar.org (J.C.); 3 Agronomy Department, University of Florida, Gainesville, FL 32611, USA; jhernandezjarqui@ufl.edu; 4 Department of Statistics, University of Nebraska-Lincoln, Lincoln, NE 68583, USA; rekahoward@unl.edu; 5 Lethbridge Research and Development Centre, Agriculture and Agri-Food Canada, 5403 1st Avenue South, Lethbridge, AB T1J 4B1, Canada; brian.beres@agr.gc.ca

**Keywords:** breeding, LPSI, multi-trait selection, organic agriculture, Prairie provinces, RLPS, selection index, Smith index

## Abstract

Both the Linear Phenotypic Selection Index (LPSI) and the Restrictive Linear Phenotypic Selection Index (RLPSI) have been widely used to select parents and progenies, but the effect of economic weights on the selection parameters (the expected genetic gain, response to selection, and the correlation between the indices and genetic merits) have not been investigated in detail. Here, we (i) assessed combinations of 2304 economic weights using four traits (maturity, plant height, grain yield and grain protein content) recorded under four organically (low nitrogen) and five conventionally (high nitrogen) managed environments, (ii) compared single-trait and multi-trait selection indices (LPSI vs. RLPSI by imposing restrictions to the expected genetic gain of either yield or grain protein content), and (iii) selected a subset of about 10% spring wheat cultivars that performed very well under organic and/or conventional management systems. The multi-trait selection indices, with and without imposing restrictions, were superior to single trait selection. However, the selection parameters differed quite a lot depending on the economic weights, which suggests the need for optimizing the weights. Twenty-two of the 196 cultivars that showed superior performance under organic and/or conventional management systems were consistently selected using all five of the selected economic weights, and at least two of the selection scenarios. The selected cultivars belonged to the Canada Western Red Spring (16 cultivars), the Canada Northern Hard Red (3), and the Canada Prairie Spring Red (3), and required 83–93 days to maturity, were 72–100 cm tall, and produced from 4.0 to 6.2 t ha^−1^ grain yield with 14.6–17.7% GPC. The selected cultivars would be highly useful, not only as potential trait donors for breeding under an organic management system, but also for other studies, including nitrogen use efficiency.

## 1. Introduction

The conventional management system uses a high quantity of synthetic chemical fertilizers, herbicides, and insecticides, while an organic management system prohibits the use of such synthetic chemicals [1]. Chemical fertilizers consist of a high proportion of nitrogen (N), which is a major nutrient to increase grain yield and grain protein content (GPC). However, it also increases production costs for wheat growers and causes environmental and health risks, including soil acidification, N leaching in groundwater, and emissions of nitrous oxide (N_2_O) that contribute to global warming [2]. As a result, the global demand for organic products has been continuously growing over the years. However, over 95% of organic production is estimated to be based on crop varieties (cultivars) that were bred for the conventional management system, which lack important traits required specifically under a low-input organic system [3]. For example, organic farmers and food processors need cultivars with better weed suppression ability and sensitivity, nitrogen-use efficiency (NUE), rhizosphere competence to suppress soil- and seed-borne diseases, and tolerance to mechanical weed control [3,4,5,6]. In wheat, most semi-dwarf cultivars, specifically bred for the conventional management system have reduced root systems that make them very dependent on high nitrogen fertilizers to attain satisfactory GPC, and they are weak in competing against weeds [7]. Taller cultivars exhibit better competitive ability against weeds than the shorter ones, due to their better light interception that directly alters photosynthetic activity in the plants [8,9,10], but they may be susceptible to lodging that reduces grain yield. Some studies proposed selecting short-statured cultivars that have erect leaves with higher leaf area to maximize light interception and increase photosynthetically active radiation, biomass, and tillering capacity [4,11,12,13].

The five breeding methods employed to develop cultivars for organic agriculture are the following: (i) indirect selection under conventional management, (ii) direct selection under organic management in all generations, (iii) selection under conventional management in early generations, followed by selection under organic management in advanced generations, (iv) marker-assisted selection (MAS), and (iv) genomic selection [4,14,15]. The University of Alberta Wheat Program, Edmonton, AB, has studied the pros and cons of these methods in diverse spring wheat lines and cultivars evaluated under both conventional and organic management systems, including comparing yield components [16,17,18], the performance of sole crop with mixtures [19,20], weed and nutrient competitive abilities [8,9,21], breadmaking quality [22], mapping genes and quantitative trait loci (QTL) associated with agronomic traits [23,24,25,26,27,28,29], and comparing the prediction accuracies of different genomic selection models [30,31]. Recently, we reported the physical positions of 44 QTLs associated with heading, flowering, and maturity [26] and 152 QTLs associated with nine agronomic and end-use quality traits in four recombinant inbred line (RIL) populations, which were evaluated under conventional and organic management systems [25]. However, only 22% of the QTLs were detected in both management systems, and the remaining QTLs were detected either in the conventional (48%) or organic (30%) management systems [25,26], which is likely due to the differential expression of genes and QTLs associated with nitrogen uptake and metabolism [32,33,34]. Hence, the management specificity of most QTLs restricts the use of the QTL information for marker-assisted selection (MAS) to develop improved cultivars for both management systems. On the other hand, both single-trait and multi-trait genome-wide prediction models revealed no statistically significant differences (*p* < 0.05) between the two management systems [30,31]. The lack of significant differences in prediction accuracies between the two management systems across seven agronomic traits recorded in three populations, and the moderate to high accuracies obtained for most traits, regardless of the management systems, provide breeders with an opportunity to use phenotypic data generated in one management for predicting the performance of lines in another management. However, the likelihood of success in any conventional or modern breeding program depends on the choice of appropriate parental combinations for initiating new crosses, which determines the genetic variation on which selection will act [35]. For breeding under low N growing environments, the choice of parents with high grain yield potential and GPC with early maturity time and short plant height are of paramount importance, which forms the basis of the present study.

Several studies have reported the advantages of simultaneously selecting for multiple traits using linear phenotypic selection indices that predict a linear function of breeding values in diverse crops, including wheat [36,37,38,39,40,41]. The selection indices include the Smith Linear Phenotypic Selection Index (LPSI) [42], the Kempthorne and Nordskog Restrictive Linear Phenotypic Selection Index (RLPSI) [43], the Eigen Selection Index Method (ESIM) [44], the Restrictive Eigen Selection Index Method (RESIM) [45], linear genomic selection index (LGSI) [46], molecular eigen selection index method (MESIM), and predetermined proportional gain eigen selection index method (PPG-ESIM). As reviewed recently by Cerón-Rojas and Crossa [47], the choice of the right selection indices depends on the type of data (phenotype alone, molecular data alone, genomic estimated breeding value, both phenotype, and molecular markers), the nature of the economic weights (fixed and known vs. fixed and unknown), and imposing restrictions (constrained vs. unconstrained). The aim, statistical theory, methodology, and pros and cons of each linear selection index have been extensively discussed by different authors [45,47,48,49,50,51,52,53,54,55]. The primary goals of selection indices are to maximize the selection response, the expected genetic gains per trait (or multiple traits), and the correlation between an index and the net genetic merit (H), which help breeders in selecting superior parents for the next generation (cycle). The selection response refers to the mean of the progeny of the selected parents, whereas the expected genetic gain per trait is the mean of the population under selection [50]. Each index is defined as a linear combination of either the observed mean phenotypic values of the traits of interest with the trait’s economic weights predefined by breeders, or both phenotype and genomic (molecular markers) data [50,56]. Selection indices have been used in several species, including wheat [40,57,58,59,60]. Most breeders, however, do not routinely use selection indices, due to the need for *a priori* knowledge of fixed effects, variance–covariance matrices, and relative economic weights [55,61]. When two or more traits are used, the assignment of an optimum economic weight for each trait has been cited as the major bottleneck for utilizing selection indices [62,63,64], which may reflect the relative economic value (market situation), preferences, retrospective results, generation interval, etc. [61].

In the current study, we chose the Smith LPSI index [42] and the RLPS to select trait donors based on the selection response, the expected genetic gain per trait, and the correlation between each index and the genetic merit. The RLPSI represents a constrained index that imposes restrictions on the expected genetic gain of a specific trait while other traits either increase or decrease without any restrictions. The objective of the RLPSI is to enhance genetic change in some traits freely, by restricting the expected changes in the other traits to zero [65,66]. The LPSI, on the other hand, represents an unconstrained index that does not impose restrictions on the expected genetic gain of any trait. Both methods require a predefined economic weight but differ in terms of restricting specific traits. There has been inconsistent reporting regarding relative economic weights [63], with some authors using no economic weights at all, some replacing economic weights with “desired gains” [67], while others assign relative economic weights either randomly [62] or using an algorithm [61], which forms another basis in the present study. The objectives of the present study were, therefore, to (i) determine the best economic weight combinations using four agronomic traits (maturity, plant height, grain yield, and GPC), (ii) compare single-trait selection and multi-trait selection indices (LPSI vs. RLPSI) with and without imposing restrictions on the expected genetic gain on grain yield or GPC, and (iii) select a subset of the top ~10% of the cultivars to serve as trait donors in developing improved germplasm for production under an organic management system.

## 2. Materials and Methods

### 2.1. Phenotyping

The present study was conducted on 196 cultivars consisting of 176 historical and modern spring wheat cultivars registered in western Canada between 1905 and 2018, and 20 unregistered advanced breeding lines (Table S1), which all, hereinafter, are referred to as cultivars. Of the 196 cultivars, 192 represented eight of the wheat classes in western Canada, originating from 14 breeding programs (institutions), and having been used in previous studies for molecular diversity and population structure analysis using the wheat 90K iSelect array [68], genome-wide association mapping [27], and as one of the populations in genomic selection [30,69]. The germplasm was evaluated for days to maturity, plant height, grain yield, and GPC at five conventionally (2017–2021) and at four organically (2018–2021) managed environments at the Crop Research Facility of the University of Alberta South Campus, Edmonton, Alberta, Canada. Each cultivar was planted in a 3.0 m × 1.14 m plot at a rate of 300 seeds m^−2^, with six rows of 19 cm spacing using a randomized incomplete block design with two replications. The number of replicates per trial in other studies varied between one and six depending on the spatial heterogeneity of the experimental field, the type of germplasm, trait complexity, and the number of environments (sites × years combinations), although two replicates provided sufficient data in genetically homogenous cultivar [70,71]. Trials were planted around mid-May of every year and harvested between the end of August and mid-September. The conventional management system received the locally recommended fertilizers and herbicides. The organic management system received 37 tons of compost per hectare following the wheat harvest but it was not given any chemical fertilizer or herbicide. The conventional land follows a four-year crop rotation of barley (*Hordeum vulgare*), canola (*Brassica napus* L.), field pea (*Pisum sativum*), and wheat, whereas the organic land followed a three-year rotation of barley, field pea, and wheat. The number of days to physiological maturity was recorded when 50% of the peduncles in a plot turned yellow, whereas plant height (cm) was measured from the base of the plants in the middle of the plot to the tip of the heads, excluding awns, at physiological maturity. Grain yield per plot was weighed after drying the grains to a moisture content of 13.5%, which was then used to estimate yield per hectare. Grain protein content (%) was estimated using SpectraStar^TM^ 2500 Near Infrared Reflectance (NIR) Spectroscopy (Unity Scientific Asia Pacific, Blaxland, Australia).

### 2.2. Data Analyses

Best linear unbiased estimators (BLUE), and variance component analyses were computed for each trait (days to maturity, plant height, grain yield, and grain protein content) using the linear mixed model implemented in Multi Environment Trial Analysis with R (META-R) v.6.04 [72]. The analyses were computed separately for agronomic traits recorded under conventional management (Set-1), organic management (Set-2), and in all environments regardless of the management systems (Set-3), as described in a previous study [31]. Genetic and phenotypic covariance matrices were computed using RindSel, an R package developed to compute phenotypic and molecular selection indices [56]. The RLPSI [43] analysis was done using BLUEs in Set-3 computed from all environments regardless of the management systems, the genetic and phenotypic covariance matrices, and the economic weights assigned to each trait. We used a total of 2304 combinations of economic weights that involved days to maturity and plant height (−1, −5, −10 and −15 for each trait), GPC (1 to 80 at an interval of 10), and grain yield (from 1 to 150 with an interval of 10). Because of the need for developing early maturing and short plants, negative economic weights were assigned for both traits. The initial analyses were done twice by imposing restrictions equal to zero on the expected genetic gain for GPC or grain yield while the other three traits either increased or decreased without any restrictions. RLPSI is useful when there is a need to impose restrictions on one or more traits, which is the case when desirable traits are negatively correlated, highly quantitative (complex), and hard to simultaneously improve. Grain yield and GPC are well known examples of negatively correlated traits [73,74], which are critical in determining the profitability of wheat production. Such types of traits require either selecting genotypes with high grain yield by keeping GPC at a desirable level, or selecting high GPC genotypes with acceptable levels of grain yield [43,75].

We used analysis of variance (ANOVA) to compare the economic weights based on the expected genetic gains, response to selection, and correlation between the index and genetic merit. For each restriction, the best five economic weights were selected based on the highest expected genetic gain for grain yield and GPC, the lowest genetic gain for maturity and plant height, the highest selection responses, and correlations between the index and the net genetic merits in all traits. The best 5 economic weights were used to analyze the phenotype data recorded under the conventional (Set-1) and organic (Set-2) management systems, respectively, using both the RLPSI and the LPSI [42]. In contrast to RLPSI, LPSI does not restrict any trait. The top 10% of the entries (20 cultivars) that had the highest index were chosen, and their means were compared with the population to compute selection response, expected genetic gain per trait, and the correlation between the index and the net genetic merit (r). The LPSI and RLPSI indices were computed in R for Windows X64 v3.6.1 using the codes summarized in Table S2. Pearson correlations, linear regression analyses, different types of graphs, and analysis of variance (ANOVA) were conducted using JMP statistical discovery software [76] v16.

### 2.3. Theory of Selection Indices and Selection Parameters

The net genetic merit: The statistical theory of linear selection indices has been reviewed elsewhere [47,50,51]. Briefly, the net genetic merit of the $i^{th}$ (i =  1, 2,…, n; n =  the number of individuals) line (H) can be written as

(1) $$H_{i}\;=\;w^{\prime }{g^{\prime }}_{i}$$

where $w^{\prime }\;=\;\left[\begin{array}{cccc} w_{1} & w_{2} & \ldots & w_{t} \end{array}\right]$ is a vector 1 × t (t =  number of traits) of known and fixed economic weights and $g_{i}^{\prime }\;=\;\left[\begin{array}{cccc} g_{i1} & g_{i2} & \ldots & g_{it} \end{array}\right]$ is a vector of true unobservable genotypic random values for t traits, with multivariate normal distribution, null expectation, and covariance matrix C. The variance of $H_{i}$ is denoted as $\sigma_{H}^{2}\;=\;w^{\prime }Cw$, with **Cw** being a vector of covariance between $H_{i}$ and vector yi.

The linear phenotypic selection index (LPSI): The LPSI for the $i^{th}$ (i =  1, 2,…, n) line can be written as

(2) $$I_{i}\;=\;\beta^{\prime }y_{i},$$

where $\beta^{\prime }\;=\;[\begin{array}{cccc} \beta_{1} & \beta_{2} & \ldots & \beta_{t} \end{array}]$ is the vector of LPSI coefficients, and ${y^{\prime }}_{i}\;=\;[\begin{array}{cccc} Y_{i1} & Y_{i2} & \ldots & Y_{it} \end{array}]$ is a vector of phenotypic values for t traits, with multivariate normal distribution, null expectation, and covariance matrix P. The variance of $I_{i}$ is denoted as $\sigma_{I}^{2}\;=\;\beta^{\prime }P\beta$.

The LPSI selection response and expected genetic gain per trait: The LPSI selection response (R) and expected genetic gain per trait (E), are, respectively,

(3) $$R\;=\;k\sigma_{H}\rho_{HI}$$

and

(4) $$E\;=\;k\frac{C\beta }{\sigma_{I}}$$

where *k* is the selection intensity, $\sigma_{H}$ and $\sigma_{I}$ are the standard deviation of H and LPSI, respectively, and $\rho_{HI}\;=\;\frac{w^{\prime }C\beta }{\sqrt{w^{\prime }Cw}\sqrt{\beta^{\prime }P\beta }}$ is the correlation between H and LPSI. All the other parameters of Equations (3) and (4) were defined earlier. Note that R is a scalar, whereas E is a vector t × 1 (t =  number of traits) of the expected value of each trait in each selection cycle. In addition, while R is the expectation of H, E is the expectation of g given the LPSI values.

Constrained (restricted) LPSI: Let $d^{\prime }\;=\;[\begin{array}{cccc} d_{1} & d_{2} & \cdots & d_{r} \end{array}]$ be a vector r × 1 of the predetermined proportional gains and assume that $\mu_{q}$ is the population mean of the *q*th trait before selection. One objective could be to change $\mu_{q}$ to $\mu_{q}\;+\;d_{q}$, where $d_{q}$ is a predetermined change in $\mu_{q}$. Let $D^{\prime }\;=\;\left[\begin{array}{ccccc} d_{r} & 0 & \cdots & 0 & -d_{1}\\ 0 & d_{r} & \cdots & 0 & -d_{2}\\ \vdots & \vdots & \ddots & \vdots & \vdots \\ 0 & 0 & \cdots & d_{r} & -d_{r-1} \end{array}\right]$ a Mallard [54] matrix (r−1) × r of predetermined proportional gains, where $d_{q}$ (q = 1,2…,r) is the qth element of vector d. In addition, let $U^{\prime }$ be a matrix of 1′s and 0′s, where 1 indicates that the traits are restricted and 0 that the traits are not restricted [43,47] and let $M^{\prime }\;=\;D^{\prime }\Psi^{\prime }$ be the Mallard [77] matrix of predetermined restrictions, where $\Psi^{\prime }\;=\;U^{\prime }C$. The constrained LPSI vector of coefficients that maximizes the response to the selection of the indices and the expected genetic gains is

(5) b = Kβ

where $K\;=\;[I_{t}-Q]$, $Q\;=\;P^{-1}M{\left(M^{\prime }P^{-1}M\right)}^{-1}M^{\prime }$ and $I_{t}$ is an identity matrix of size t × t. When D = U, the above equation is the null restricted LPSI (RLPSI) vector of coefficients [43], and when D = U and $U^{\prime }$ is a null matrix, b = β, the LPSI vector of coefficients. Thus, the constrained LPSI is the most general and it includes the LPSI as a particular case.

## 3. Results

### 3.1. Phenotypic Variation

**Figure 1:**
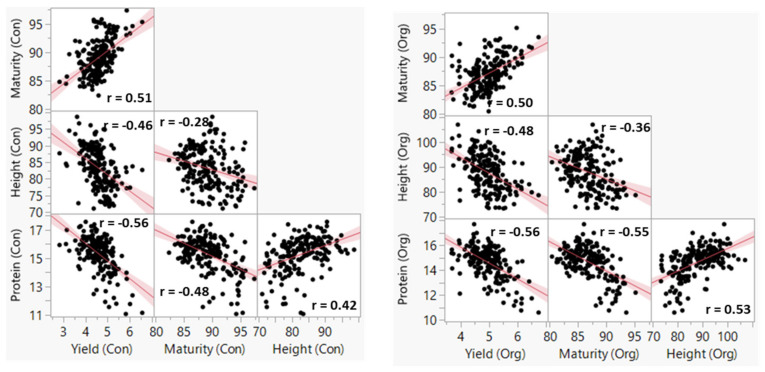
Scatter plots of the best linear unbiased estimators (BLUEs) of four agronomic traits computed from five conventionally and four organically managed environments. All correlations were significant at *p* < 0.01. The units of measurement were as follows: grain yield (t ha^−1^), plant height (cm), maturity (d), and grain protein content (%).

**Figure 2:**
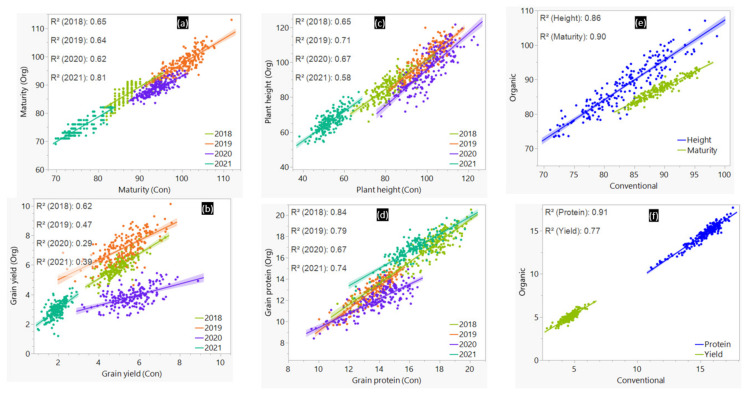
**Linear** regression plots of the best linear unbiased estimators (BLUEs) computed for each environment (**a**–**d**) and all environments within the conventional (Con) or organically (Org) management systems (**e**,**f**). The units of measurement were as follows: grain yield (t ha^−1^), plant height (cm), maturity (**d**), and grain protein content (%).

The maturity time, plant height, grain yield, and GPC of the BLUEs computed per management varied from 81 to 97 days, from 71 to 107 cm, from 2.8 to 6.7 t ha^−1^, and from 10.6 to 17.7%, respectively (Table S1). As shown in Figure 1, grain yield recorded in the two management systems showed significant (*p* < 0.01) moderate negative correlation with both GPC (−0.56) and plant height (−0.48 ≤ r ≤ −0.46), but positive correlation with maturity (0.50 ≤ r ≤ 0.51). The coefficients of determination (R^2^) between pairs of environments under the conventional and organic management systems varied from 0.62 to 0.81 for maturity, from 0.58 to 0.71 for plant height, from 0.29 to 0.62 for grain yield, and from 0.67 to 0.84 for GPC (Figure 2). When BLUEs computed from all environments per management were used instead of the individual environments in the scatter plots, R^2^ was high to very high for GPC (0.91), maturity (0.90), and plant height (0.86), and grain yield (0.77). Cultivars, environments, and GE interactions had a significant (*p* < 0.001) effect in the model in both management systems and all traits (data not shown). Broad-sense heritability computed across all environments within the conventional and organic management systems varied from 0.43 to 0.80 and from 0.32 to 0.60, respectively (Table 1).

### 3.2. Single Trait Selection

Using a selection intensity of 10%, we first selected the top 20 cultivars that produced the highest grain yield in each management system regardless of maturity, plant height, and GPC. In both management systems, a total of 27 cultivars were selected based on grain yield alone (Table S3), of which 13 cultivars (48.1%) produced high grain yield in both management systems (5702PR, AAC Awesome, AAC Goodwin, AC Andrew, Bhishaj, CDC Throttle, Faller, Fielder, GP168, Pasteur, SAAR, Sadash, and SWS-52). The remaining 14 cultivars produced high yield either in the conventional (7) or organic (7) management. We then selected the same number of cultivars based on GPC regardless of the other three traits, which identified a total of 26 cultivars. Fourteen of the 26 cultivars (53.8%) were common in both managements (AC Barrie, AC Cadillac, AC Eatonia, BYT14-19, CDC VR Morris, Jake, Lancer, Leader, Prodigy, PT771, Roblin, Somerset, SY479 VB, and SY637). The remaining cultivars were selected based on their high GPC either in the conventional (5605HR CL, AAC Castle, AC Cora, Burnside, BW278, and Parata) or organic (CDC Alsask, CDC Bounty, Lillian, Lovitt, Pasqua, and PT472) management system. None of the cultivars selected based on grain yield were common with those selected based on GPC, which is not surprising, due to the negative correlations between the two traits.

### 3.3. Comparisons of Economic Weights

**Figure 3:**
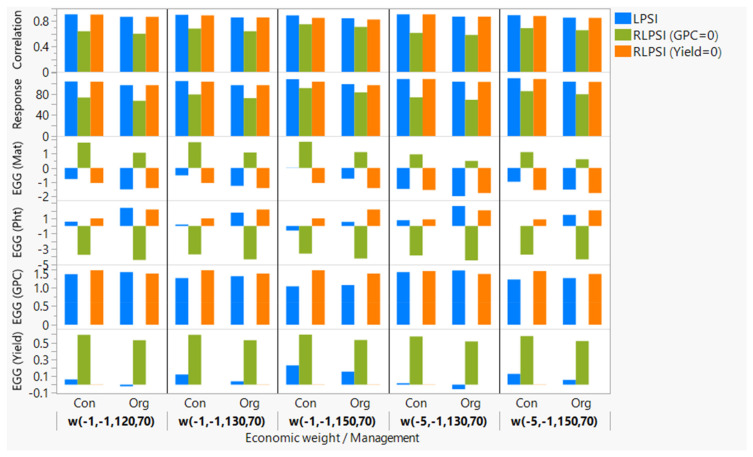
Bar graph of expected genetic gain (EGG) for grain yield (Yield), plant height (Pht), maturity (Mat), and grain protein content (GPC) plus response to selection and the correlations between each index and the genetic merit based on the Smith Linear Phenotypic Selection Index (LPSI) and Kempthorne and Nordskog Restrictive Linear Phenotypic Selection Index (RLPSI). The RLPSI analyses were done by restricting the expected genetic gain either for yield or GPC to zero. The best linear unbiased estimators computed from the conventionally or organically managed environments and five economic weights (w) were used in the analysis. The economic weights, such as w(−1, −1, 120, 70) refer to an economic weight of −1 for maturity, −1 for plant height, 120 for grain yield, and 70 for GPC, respectively. See Table S6 for details.

The identification of optimal economic weights is the prerequisite for multi-trait selection using LPSI and RLPS. For such a purpose, we performed RLPSI analyses using BLUEs computed from all environments regardless of the management system (Set-3) and the 2304 combinations of economic weights. The analyses provided highly variable expected genetic gains, response to selection, and correlation between the index and genetic merit. The genetic gains computed from all 2304 economic weights varied from −4.5 to 1.5 d for maturity, from −13.0 to 2.0 cm for plant height, from −0.25 to 0.65 t ha^−1^ for grain yield, and from −0.50 to 1.60% for GPC (Figure S1, Table S4). When ANOVA was performed to compare differences among economic weights within each trait (4 weights for maturity and plant height each, 9 for GPC, and 16 for yield), we found significantly greater (*p* < 0.01) genetic gain for grain yield when the weights were set between 120 and 150 with −1 or −5 weights for both maturity and plant height regardless of the economic weight for GPC (Table S5). Forty-eight of the 2304 economic weights (Figure 3) gave the highest expected genetic gain for yield (0.63–0.65 t ha^−1^), maturity (<1.5 d), plant height of (<−3.8 cm), selection response (>80), and correlation coefficients between the index and the genetic merit (≥0.90). ANOVA performed on the genetic gains obtained after imposing restrictions on the expected genetic gain on grain yield showed a significantly greater genetic gain for GPC when weights were set to 70 and 80 for GPC and −1 for plant height, regardless of the weights both for maturity and grain yield. Sixteen of the 2304 economic weights that consisted of maturity (−1 and −5), plant height (−1), yield (120–150), and GPC (70 and 80) showed the highest expected genetic gain for GPC (1.56–1.58%), maturity (<–1.5 d), plant height of (<2.0 cm), selection response (>80), and correlation coefficients between the index and the genetic merit (≥0.90). Based on the results of the two analyses, we selected the following five economic weights (−1, −1, 120, 70 vs. −1, −1, 130, 70 vs. −1, −1, 150, 70 vs. −5, −1, 130, 70 vs. −5, −1, 150, 70) corresponding to maturity, plant height, yield, and GPC, respectively, for final analyses using both RLPSI and LPSI.

### 3.4. Multi-Trait Selection Using RLPSI by Restricting Grain Protein Content

The expected genetic gain for grain yield estimated using the five economic weights after restricting GPC varied from 0.57 to 0.59 t ha^−1^ in the conventional, and from 0.51 to 0.53 t ha^−1^ in the organic, management systems (Table 1). The gains in days to maturity, plant height, response to selection, and correlation coefficients in both management systems varied from 0.49 to 1.82 d, from −4.49 to 3.61 cm, from 66.74 to 90.90, and from 0.58 to 0.75, respectively. Using a selection intensity of the top 10%, we selected a total of 30 of the 196 cultivars (Table S7) of which 18 were identified using all five economic weights under the organic management systems. However, only 10 of the 30 selected cultivars (AAC Awesome, AAC Brandon, AAC Castle, AAC Goodwin, AAC Penhold, AAC Viewfield, CDC Throttle, Conquer, Faller, and SWS-52) were consistently selected using all five economic weights in both management systems. Overall, the 30 selected cultivars in the two management systems required 85–97 d to maturity, were 72–96 cm tall, and produced 4.8–6.7 t ha^−1^ grain yield with 10.6–16.7% GPC regardless of the economic weights and management systems (Table S7). Days to maturity, plant height, grain yield, and GPC in three check cultivars (AAC Viewfield, Carberry, and Glenn) that are widely used in western Canada varied from 86 to 97 d, from 74 to 85 cm, from 3.9 to 5.6 t ha^−1^, and from 14.7 to 15.3%, respectively, regardless of the management systems. As compared with the checks, some of the selected cultivars were taller and/or had lower GPC, which was expected, due to differences in the market classes. The checks belong to the Canada Western Red Spring (CWRS) class, but the selected cultivars belong to the CWRS (12 cultivars), Canada Prairie Spring Red (CPSR) (8), Canada Northern Hard Red (CNHR) (5), Canada Western Special Purpose (CWSP) (3), Canada Western Extra Strong (CWES) (1) and Canada Western Soft White Spring (CWSWS) (1) classes. Days to maturity, plant height, grain yield, and GPC of the 12 selected CWRS cultivars (AAC Brandon, AAC Elie, AAC Viewfield, AAC Redberry, CDC Landmark, BW5020, CDC Imagine, CDC Hughes, Stettler, Superb, SY 433, and Zealand) varied from 85–94 days, 72–96 cm, 4.8–5.6 t ha^−1^, and 14.4–16.3%, respectively.

### 3.5. Multi-Trait Selection Using RLPSI by Restricting Grain Yield

The expected genetic gain estimated for GPC using the five economic weights was 1.5% in the conventional and 1.4% in the organic management systems (Table 1). The gain in maturity, plant height, and grain yield across the five economic weights in the two management systems varied from −1.7 to 1.0, from 0.9 to 2.2 cm, from 1.4 to 1.5%, and zero, respectively. The response to selection and correlation between the index and genetic merit ranged from 96.3 to 108.3 and from 0.83 to 0.91, respectively (Table 1). Using a 10% selection intensity, we identified a total of 31 of the 196 cultivars based on GPC, maturity, and plant height of which 20 were identified using all five economic weights under the organic management systems. The 31 selected cultivars belong to the CWRS (24 cultivars), CPSR (2), CNHR (3), and CWES (2), and required 83–93 d to maturity, were 73–100 cm tall, and produced 4.0–6.2 t ha^−1^ grain yield with 14.6–17.7% GPC (Table S6). However, only 10 of the 31 cultivars (5605HR CL, AAC Brandon, AAC Goodwin, AAC Tisdale, AC Cadillac, BYT14-19, CDC VR Morris, Conquer, Somerset, and SY479 VB) were consistently selected using all five economic weights in both management systems (Table S7).

### 3.6. Multi-Trait Selection Using LPSI

The phenotypic performance and expected genetic gains were significantly different between the LPSI and RLPSI (Figure 3). In both management systems, the expected genetic gains for maturity, plant height, grain yield, and GPC obtained using the LPSI without imposing any restriction and the five economic weights varied from −2.0 to 0 d, from −0.6 to 2.6 cm, from −0.1 to 0.2 t ha^−1^, and from 1.0 to 1.5%, respectively. The response to selection and correlation between the index and genetic merit ranged from 96.4 to 109.8 and from 0.84 to 0.91, respectively (Table 1). The LPSI selected 35 of the 196 cultivars (Table S7) of which 17 cultivars (5605HR CL, AAC Brandon, AAC Goodwin, AAC Viewfield, AC Cadillac, CDC Alsask, CDC Bounty, CDC Imagine, CDC Thrive, CDC VR Morris, Conquer, Goodeve, Lillian, Lovitt, PT472, Somerset, and Zealand) were chosen using all five economic weights under the organic management system. All these 17 cultivars were also selected using RLPSI by restricting grain yield. However, only 5 of 35 cultivars (5605HR CL, AAC Brandon, AAC Goodwin, CDC VR Morris, and Conquer) were selected using all five economic weights in both management systems. The selected cultivars belong to the CWRS (24), CPSR (5), CNHR (3), and CWES (3), and required 83–94 d to maturity, were 72–100 cm tall, and produced 4.2 t ha^−1^ grain yield with 14.6–17.7% GPC (Table S7).

### 3.7. Comparison of Economic Weights and Selection Indices

**Figure 4:**
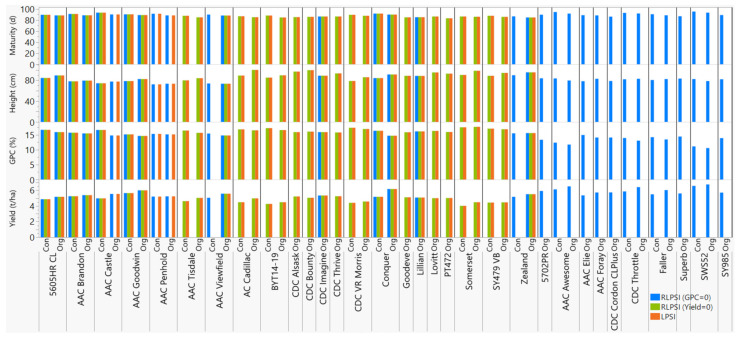
Comparison of the phenotypic performance of 32 cultivars consistently selected for their superior performance under organic management system using all five economic weights and the Smith Linear Phenotypic Selection Index (LPSI) and Kempthorne and Nordskog Restrictive Linear Phenotypic Selection Index (RLPSI). The RLPSI analyses were done by restricting the expected genetic gain either for yield (Yield = 0) or grain protein content (GPC = 0). The y-axes represent the best linear unbiased estimators computed from the conventionally or organically managed environments. See Table S1 for details.

Overall, the three selection indices (LPSI plus RLPSI by restricting grain yield and GPC) identified a total of 47 cultivars that performed very well under the conventional and/or organic management, of which 22 cultivars were consistently selected using all five economic weights and at least two of the three selection scenarios (Table 2 and Table S7). Six of the 22 cultivars (AAC Brandon, AAC Goodwin, AAC Viewfield, CDC Imagine, Conquer, and Zealand) were selected using all five economic weights and three scenarios, while 11 of the cultivars (5605HR CL, AC Cadillac, CDC Alsask, CDC Bounty, CDC Thrive, CDC VR Morris, Goodeve, Lillian, Lovitt, PT472, and Somerset) were selected using both the LPSI and RLPSI by restricting grain yield. The remaining 5 cultivars (AAC Castle, AAC Penhold, AAC Tisdale, BYT14-19, and SY479 VB) were selected using all five economic weights in the RLPSI index by restricting grain yield plus the LPSI (Table 2, Figure 4). We also compared these 22 selected cultivars against the 53 cultivars (Table S3) selected using either grain yield or GPC regardless of the other traits, which revealed 15 common cultivars between the single trait selection and multi-trait selection methods. The latter included 5605HR CL, AAC Brandon, AAC Castle, AAC Goodwin, AC Cadillac, BYT14-19, CDC Alsask, CDC Bounty, CDC VR Morris, Conquer, Lillian, Lovitt, PT472, Somerset, and SY479 VB.

## 4. Discussion

Selection indices have been used in plant breeding for the simultaneous improvement of more than one trait [78,79,80]. The LPSI method proposed by Smith is one of the simplest indices that showed better performance for simultaneously improving two or more traits than the independent culling and tandem selection [81]. The index-based selection was found to be particularly useful for negatively correlated traits that are hard to simultaneously improve [82], which was the case between grain yield and GPC, yield and plant height, and maturity and GPC in both conventional and organic management systems (Figure 1). In such cases, phenotypic selection indices have been used for simultaneously improving multiple negatively correlated traits in wheat, including grain yield and GPC [83], spot blotch resistance, early maturity, and short plant height [60], Helminthosporium leaf blight resistance, early maturity, grain yield, and kernel weight [82]. In oat, Dolan et al. [84] reported the superiority of both the restricted index and Smith index, as compared with selection for grain yield alone, for simultaneously improving heading date, plant height, and barley yellow dwarf virus resistance. In contrast to the restricted (constrained) methods, such as RLPSI, however, the LPSI method does not provide breeders with an opportunity to hold some traits constant while allowing other traits to increase freely [66]. The choice of the index affects selection response, genetic gain per trait, and the correlation between the index and the net genetic merit (r) depending on the genetic relationship among target traits [83], which was evident in the present study. As compared with the Kempthorne and Nordskog restricted RLPSI index, the Smith LPSI index gave a very small expected genetic gain for grain yield regardless of the economic weights and management systems (Figure 3). Furthermore, about a third of the 32 lines selected using the three scenarios were also selected only in the Smith index. Our results disagreed with Marulanda et al. [83] who reported between 13.5% and 112.2% greater genetic gains for grain yield and GPC in wheat using the Smith index than the Kempthorne and Nordskog restricted index.

The major challenge in the use of multi-trait phenotypic selection indices is finding optimum economic weights across traits, which reflects the relative importance of one trait to another in making up the overall value of a line. The choice of economic weights not only affects the ranks of the selected parents for the next generation, but also the expected selection response, genetic gain, and the correlation between the index and the net genetic merit [47,62,63,78]. For such reasons, we assessed the selection parameters across 2304 economic weights, which demonstrated the significant effect of weights not only in the selection parameters (Figure S1, Table S4), but also on the selected genotypes under each management system (Table S7). Although several studies have previously assessed the effect of a smaller number of economic weights, our results based on such large combinations of economic weights provide breeders with a better overview of the sensitivity of multi-trait selection indices and the need to balance the different selection parameters. Priority was given to economic weights that provided smaller expected genetic gain for maturity and plant height but greater grain for yield and GPC with a higher response to selection and correlation coefficients between the index and genetic merits [47]. Using the five best combinations of economic weights, three selection scenarios (LPSI, RLPS with GPC = 0 or Yield = 0), and two management systems, we chose a total of 16 CWRS cultivars (AAC Brandon, AAC Tisdale, AAC Viewfield, AC Cadillac, BYT14-19, CDC Alsask, CDC Bounty, CDC Imagine, CDC Thrive, CDC VR Morris, Goodeve, Lovitt, PT472, Somerset, SY479 VB, and Zealand), 3 CNHR cultivars (5605HR CL, Conquer, and Lillian), and 3 CPSR cultivars (AAC Castle, AAC Goodwin, and AAC Penhold) that could be used as donor parents for breeding under an organic management system (Figure 4, Tables S1 and S7). These 22 selected cultivars originated from the Agriculture and Agri-Food Canada (AAFC) breeding programs (9 cultivars), the University of Saskatchewan breeding program (5), the University of Alberta wheat breeding program (2), the Syngenta Canada Inc. (2), SeCan Association (2), Canterra Seeds (1), and Farm Pure Seeds (1).

AAC Brandon [85] is a CWRS cultivar developed by the AAFC researchers located at the Swift Current Research and Development Centre, Swift Current, SK, from the cross Superb/CDC Osler//ND744. AAC Brandon is characterized by short plants with a strong straw (lodging tolerant), resistance to prevalent races of leaf, stem, and stripe rust, moderate resistance to Fusarium head blight (FHB) and loose smut, and comparable with the best CWRS checks in terms of yield and maturity [85]. AAC Brandon is the most popular cultivar in Manitoba for both organic and conventional growers. In Alberta, AAC Brandon is one of the two spring wheat cultivars grown by five or more organic producers from 2017 to 2020 (https://afsc.ca/wp-content/uploads/2021/02/Yield-Alberta-2021.pdf; accessed on 7 July 2022). In both management systems in the present study, AAC Brandon required 89–91 days for maturity, was 78–80 cm tall and produced 5.2–5.4 t ha^−1^ grain yield with 15.5–15.7% GPC (Table 2, Figure 4). AAC Tisdale (PT250) is a CWRS cultivar developed by the Swift Current Research and Development Centre from the cross Somerset/BW865//Waskada. AAC Tisdale has been reported to have a medium height, high yield potential, high GPC and has been tested for commercial production under organic farming (https://www.prairieorganics.org/wheatvarietytrials; accessed on 7 July 2022). In the current study, AAC Tisdale plants were 80–84 cm tall, required 85–88 days to maturity, and produced 4.6–5.0 t ha^−1^ grain yield with 15.7–16.4% GPC (Table 2, Figure 4). AAC Viewfield [86] is a CWRS cultivar developed by the Swift Current Research and Development Centre from the cross Stettler/Glenn. As compared with check cultivars, AAC Viewfield has been reported to be high yielding, late maturing with shorter plant stature, low lodging score, resistant to prevalent races of yellow rust and stem rust, moderate resistance to leaf rust and common bunt, and intermediate resistance to FHB [86]. In the present study, this cultivar matured within 88–90 days, plants were 73–74 cm tall and produced 5.1–5.6 t ha^−1^ with 14.8–15.4% GPC (Table 2, Figure 4).

AC Cadillac [87] is a CWRS cultivar jointly developed by the AAFC researchers at the Swift Current Research and Development Centre and the Lethbridge Research Centre, Lethbridge, AB, from the cross BW90*3/BW553. It is characterized by high grain yield with high GPC, heavy kernel and volume weights, improved resistance to leaf spots, and resistance to prevalent races of leaf rust, stem rust, loose smut, and common bunt. In the present study, AC Cadillac required 85–87 days for maturity, were 89–100 cm tall, and produced 4.5–5.0 t ha^−1^ with 16.5–16.9% GPC (Table 2, Figure 4). BYT14-19 is an unregistered CWRS line developed by the University of Alberta Wheat Breeding Program from the cross Carberry/PT764//CDC Stanley and characterized by good lodging tolerance and excellent resistance against stem and stripe rusts and FHB (M. I., unpublished). In the present study, BYT14-19 plants were 85–90 cm tall, matured within 85–88 d, and produced 4.3–4.5 t ha^−1^ grain yield with 16.6–17.2% GPC (Table 2, Figure 4). CDC Alsask is a CWRS cultivar developed by the Crop Development Centre at the University of Saskatchewan, Saskatoon, SK, from the cross AC Elsa/AC Cora. In the present study, the CDC Alsask plants evaluated in the two management systems were taller (88–97 cm), required 85–88 days for maturity, and produced 4.6–5.2 t ha^−1^ grain yield with 15.9–16.4% GPC (Table 2, Figure 4). CDC Bounty (BW-720) is a CWRS cultivar developed by the Crop Development Centre at the University of Saskatchewan from the cross Katepwa/W-82624//Kenyon. In the current study, this cultivar was 90–100 cm tall, required 86 days to maturity, and produced 4.3–5.0 t ha^−1^ grain yield with 16.1–16.5% GPC (Table 2, Figure 4). CDC Imagine (BW758) is a CWRS cultivar developed by the Crop Development Centre at the University of Saskatchewan from the cross CDC Teal*4/Fidel(FS2). This cultivar is known for its resistance to the imidazolinone class of herbicides. In both the conventional and organic management systems, CDC Imagine plants required 86–88 days to maturity, were 84–89 cm tall, and produced 4.7–5.3 t ha^−1^ grain yield with 15.9–16.3% GPC. CDC Thrive (PT575) is another CWRS cultivar developed by the Crop Development Centre at the University of Saskatchewan from the cross CDC Bounty/CDC Imagine. CDC Thrive plants were 89–94 cm tall, required 86–88 days for maturity, and produced 4.8–5.2 t ha^−1^ grain yield with 15.8–16.0% GPC (Table 2, Figure 4). CDC VR Morris (BW423) is also a CWRS cultivar developed by the Crop Development Centre at the University of Saskatchewan from the cross CDC Bounty/FHB9 characterized by high yield, high GPC, heavy test weight, strong lodging resistance, resistant to leaf rust, intermediate resistant to leaf spot, moderate resistance to both stem rust and FHB (https://provenseed.ca/cereals/cdcvrmorris; accessed on 7 July 2022). In our study, the CDC VR Morris plants matured within 88–90 days, were 79–86 cm tall, and produced 4.4–4.6 t ha^−1^ grain yield with 17.0–17.4% GPC (Table 2, Figure 4).

Goodeve [88] is a CWRS cultivar developed by the Swift Current Research and Development Centre from the cross 94B43-BLW4/AC Intrepid. In previous studies, this cultivar was characterized by high grain yield, early maturity, shorter plant height, lodging tolerance, higher GPC, resistance to prevalent races of stem rust and loose smut, moderate resistance to leaf rust, resistance to the insect orange blossom wheat midge, moderate susceptibility to common bunt, and susceptible to FHB. In the current study, the Goodeve plants were 81–90 cm tall, matured within 85–86 days, and produced 4.5–5.1 t ha^−1^ grain yield with 15.9–16.0% GPC (Table 2, Figure 4). Lovitt [89] is a CWRS cultivar developed by the Swift Current Research and Development Centre from the cross 8405-JC3C*2/BW152. Lovitt is an earlier maturing cultivar with very good pre-harvest sprouting resistance and resistance to prevalent races of leaf rust, stem rust, and loose smut. In both management systems, Lovitt plants were about 87–95 cm tall, matured within 87 days, and produced 4.1–5.0 t ha^−1^ grain yield with 16.3 GPC (Table 2, Figure 4). PT472 is a CWRS unregistered line developed by the AAFC researchers at the Brandon Research and Development Centre [90] from the cross PT425/Helios//PT435 and characterized by early maturity, high grain yield and test weight, and excellent clean wheat flour yield. In the current study, PT472 plants were 87–93 cm tall, required 83–85 days for maturity, and produced 4.7–5.0 t ha^−1^ grain yield with 16.0% GPC (Table 2, Figure 4). Somerset [91] is a CWRS cultivar developed by the AAFC researchers at the Cereal Research Centre, Winnipeg, MB from the cross 90B01-AD4D/Pasqua. It is characterized by taller plants, high GPC, intermediate grain yield, resistance to stem rust, leaf rust and loose smut, resistance to FHB, and lower test weight. In the current study, Somerset plants were 91–99 cm tall, matured within 86–87 d, and produced 4.0–4.5 t ha^−1^ grain yield with 17.6–17.7% GPC (Table 2, Figure 4).

SY479 VB is a CWRS cultivar developed by the Syngenta Canada Inc. from the cross 01S2004-2-13/Glenn and characterized by an early maturity, good yield potential, high GPC, very good resistance to sprouting, resistance to common bunt and leaf rust, moderate resistance to stem rust and FHB, and poor resistance to leaf spot and loose smut. In the current study, SY479 VB plants were 89–95 cm tall, required 86–88 days to mature, and produced 4.4–4.5 t ha^−1^ grain yield with 16.9–17.1% GPC (Table 2, Figure 4). Zealand [92] is a CWRS cultivar developed by the University of Alberta Wheat Breeding Program from the cross between Alvena, a CWRS wheat cultivar (Knox et al., 2008), and FHB resistant line (IAS64/ALDAN//URES/3/TNMU/4/TNMU) from the International Maize and Wheat Improvement Center (CIMMYT). This cultivar is characterized by high yield potential, high GPC, taller plants, large leaves, early maturity, resistance to the prevalent races of leaf rust, moderate resistance to stripe rust and loose smut, intermediate resistance to stem rust and leaf spot, and moderately susceptible to common bunt and FHB. In the current study, Zealand plants were 90–96 cm tall, matured within 85–87 days, and produced 5.2–5.5 t ha^−1^ grain yield with 15.5–15.6% GPC (Table 2, Figure 4).

5605HR CL (BW918) is a CNHR cultivar developed by the Syngenta Canada Inc. from the cross 99S2232-10/99S3228-4. In both management systems, 5605HR CL plants required 88–90 days for maturity, were 85–90 cm tall, and produced 4.9–5.2 t ha^−1^ grain yield with 15.9–16.7% GPC (Table 2, Figure 4). Conquer [93] is a CNHR cultivar developed by the AAFC researchers at the Cereal Research Centre from the cross HY639/99 EPWA-Mdg 61. Conquer is characterized by good agronomic performance, high yield potential, high GPC, resistance to orange wheat blossom midge and hessian fly, good resistance to leaf rust, stem rust, stripe rust, and common bunt. In the conventional and organic management systems, the Conquer plants were 85–91 cm tall, matured within 90–92 days, and produced 5.2–6.2 t ha^−1^ grain yield with 14.7–16.4% GPC (Table 2, Figure 4). Lillian [94] is another CNHR cultivar jointly developed by the AAFC researchers at the Cereal Research Centre and the Swift Current Research and Development Centre from the cross BW621*3/90B07-AU2B. It is an early maturing and high yielding cultivar with improved GPC and resistance to leaf rust, and leaf spot. In the present study, Lillian plants were 84–89 cm tall, matured within 85–86 days, and produced 4.3–5.1 t ha^−1^ grain yield with 16.2% GPC (Table 2, Figure 4).

AAC Castle [95] is a CPSR cultivar developed jointly by the AAFC researchers at the Cereal Research Centre and the Lethbridge Research and Development Centre from the cross Conquer/CDN Bison//5701PR. It is characterized by high grain yield and GPC with excellent resistance to leaf, stem, stripe rust, common bunt and loose smut, and tolerance to the orange wheat blossom midge. In the conventional and organic management systems, AAC Castle plants were 75–78 cm tall, required 90–93 days to maturity, and produced 5.0–5.5 t ha^−1^ grain yield with 14.8–16.6% GPC (Table 2, Figure 4). AAC Goodwin (also BW968) is a CPSR cultivar developed by the Swift Current Research and Development Centre from the cross Carberry/AC Cadillac and characterized by high yield, short stature, strong straw, and medium to late maturity. In the present study, AAC Goodwin plants required 89–91 days for maturity, were 79–83 cm tall, and produced 5.6–6.0 t ha^−1^ with 14.6–15.2% GPC (Table 2, Figure 4). AAC Penhold [96] is CPSR cultivar developed by the Swift Current Research and Development Centre from the cross 5700PR/HY644-BE//HY469. It is an early maturing, short stature, and high yielding cultivar with improved GPC, resistance to prevalent races of leaf rust, common bunt, and moderate resistance to FHB and stem rust. In the present study, AAC Penhold plants were 72–74 cm tall, required 88–91 days to maturity, and produced 5.2 t ha^−1^ with 15.3% GPC (Table 2, Figure 4).

## 5. Conclusions

The multi-trait linear phenotypic selection indices, with and without restricting the expected genetic gain of a particular trait, were superior to single trait selection. However, the selection parameters (expected genetic gain per trait, the response to selection, and the correlation coefficients between each index and genetic merits) differed depending on the economic weights used for analyses. From a breeding point of view, the consistency of the subset of the top selected genotypes across economic weights and selection indices were equally important to the selection paraments. Using 2304 combinations of economic weights among four agronomic traits, we demonstrated the need for identifying an optimal combination of economic weights that not only affects the selection parameters, but also the selected cultivars. The twenty-two cultivars that showed better performance under organically managed environments consisted of 16 CWRS (AAC Brandon, AAC Tisdale, AAC Viewfield, AC Cadillac, BYT14-19, CDC Alsask, CDC Bounty, CDC Imagine, CDC Thrive, CDC VR Morris, Goodeve, Lovitt, PT472, Somerset, SY479 VB, and Zealand), 3 CNHR (5605HR CL, Conquer and Lillian), and 3 CPSR (AAC Castle, AAC Goodwin, and AAC Penhold). Some of the cultivars shared common parentages. Further studies are needed to understand the weed tolerance and nitrogen use efficiency of the 22 selected cultivars.

## Figures and Tables

**Table 1:** Broad-sense heritability, expected genetic gain (EGG) per trait, response to selection, and correlation between each index and genetic merit based on five economic weights (Wt) and three selection scenarios. The orders of traits in the economic weights were maturity, plant height, grain yield, and grain protein content, respectively. In contrast to the linear phenotypic selection index (LPSI) that did not constrain any trait, the restricted linear phenotypic selection index (RLPSI) constrained the expected genetic gain of either grain yield or GPC to zero. Mat, Pht, Yld, and GPC refer to maturity (days), plant height (cm), grain yield (t ha^−1^), and grain protein content (%), respectively.

Economic Weight	Scenarios	EGG (Mat)		EGG (Pht)		EGG (Yld)		EGG (GPC)		Response		Correlation	
		Con	Org	Con	Org	Con	Org	Con	Org	Con	Org	Con	Org
Heritability		0.51	0.38	0.43	0.58	0.48	0.32	0.80	0.60				
Wt (−1, −1, 120, 70)	LPSI	−0.78	−1.49	0.58	2.37	0.06	−0.02	1.37	1.43	103.41	96.35	0.91	0.87
	RLPSI (GPC = 0)	1.77	1.07	−3.76	−4.43	0.59	0.53	0.00	0.00	73.10	66.74	0.64	0.60
	RLPSI (Yield = 0)	−1.05	−1.40	0.99	2.16	0.00	0.00	1.47	1.39	103.08	96.31	0.91	0.87
Wt (−1, −1, 130, 70)	LPSI	−0.52	−1.24	0.18	1.76	0.12	0.04	1.26	1.31	104.32	96.44	0.90	0.86
	RLPSI (GPC = 0)	1.79	1.08	−3.70	−4.36	0.59	0.53	0.00	0.00	79.03	72.02	0.68	0.64
	RLPSI (Yield = 0)	−1.05	−1.40	0.99	2.16	0.00	0.00	1.47	1.39	103.08	96.31	0.89	0.86
Wt (−1, −1, 150, 70)	LPSI	−0.01	−0.75	−0.58	0.54	0.23	0.15	1.04	1.07	107.82	98.37	0.89	0.84
	RLPSI (GPC = 0)	1.82	1.11	−3.61	−4.24	0.59	0.53	0.00	0.00	90.90	82.61	0.75	0.71
	RLPSI (Yield = 0)	−1.05	−1.40	0.99	2.16	0.00	0.00	1.47	1.39	103.08	96.31	0.85	0.83
Wt (−5, −1, 130, 70)	LPSI	−1.46	−1.96	0.76	2.61	0.02	−0.06	1.42	1.47	108.30	102.90	0.91	0.87
	RLPSI (GPC = 0)	0.96	0.49	−3.83	−4.49	0.57	0.51	0.00	0.00	73.47	68.84	0.62	0.58
	RLPSI (Yield = 0)	−1.54	−1.75	0.87	2.05	0.00	0.00	1.45	1.37	108.27	102.63	0.91	0.87
Wt (−5, −1, 150, 70)	LPSI	−0.96	−1.51	0.00	1.47	0.13	0.06	1.23	1.26	109.76	102.92	0.89	0.86
	RLPSI (GPC = 0)	1.11	0.60	−3.73	−4.36	0.58	0.52	0.00	0.00	84.98	79.17	0.69	0.66
	RLPSI (Yield = 0)	−1.54	−1.75	0.87	2.05	0.00	0.00	1.45	1.37	108.27	102.63	0.88	0.85

**Table 2:** The pedigree information and summary of best linear unbiased estimators for the 22 selected cultivars and lines based on 5 conventionally (Con) and 4 organically (Org) managed environments.

Cultivar (Line)	Pedigree	Wheat Class *	Registration Year	Maturity (d)		Plant Height (cm)		Yield (t/ha)		GPC (%) **	
				Con	Org	Con	Org	Con	Org	Con	Org
5605HR CL	99S2232-10/99S3228-4	CNHR	2013	89.6	88.4	84.8	89.6	4.9	5.2	16.7	15.9
AAC Brandon	Superb/CDC Osler//ND744	CWRS	2013	91.0	88.7	78.1	79.6	5.2	5.4	15.7	15.5
AAC Penhold	5700PR/HY644-BE//HY469	CPSR	2014	91.4	88.5	72.4	73.7	5.2	5.2	15.3	15.2
AAC Viewfield	Stettler/Glenn	CWRS	2016	90.1	88.2	74.1	73.6	5.1	5.6	15.4	14.8
AC Cadillac	BW90 * 3/BW553	CWRS	1996	86.8	85.2	89.4	100.1	4.5	5.0	16.9	16.5
AAC Goodwin	Carberry/AC Cadillac	CPSR	2017	90.5	89.0	78.6	82.5	5.6	6.0	15.2	14.6
Zealand	Alvena/IAS64/ALDAN//URES/3/TNMU/4/TNMU)	CWRS	2016	86.6	84.7	90.1	95.5	5.2	5.5	15.5	15.6
BYT14-19	Carberry/PT764//CDC Stanley	CWRS	-	88.1	84.9	85.4	89.9	4.3	4.5	17.2	16.6
CDC Alsask	AC Elsa/AC Cora	CWRS	2005	87.7	85.3	88.1	96.9	4.6	5.2	16.4	15.9
CDC Bounty	Katepwa/W-82624//Kenyon	CWRS	2000	86.1	85.9	90.0	99.7	4.3	5.0	16.5	16.1
CDC Imagine	CDC Teal * 4/Fidel(FS2)	CWRS	2002	88.4	86.4	84.2	89.2	4.7	5.3	16.3	15.9
CDC Thrive	CDC Bounty/CDC Imagine	CWRS	2010	88.4	86.5	89.4	93.5	4.8	5.2	16.0	15.8
CDC VR Morris	CDC Bounty/FHB9	CWRS	2012	89.6	87.8	78.8	86.1	4.4	4.6	17.4	17.0
Conquer	HY639/99 EPWA-Mdg 61	CNHR	2010	91.7	89.9	84.5	91.4	5.2	6.2	16.4	14.7
Goodeve	94B43-BLW4/AC Intrepid	CWRS	2007	85.9	85.0	80.5	88.9	4.5	5.1	16.0	15.9
AAC Castle	Conquer/CDN Bison//5701PR	CPSR	2018	93.4	90.3	74.6	77.7	5.0	5.5	16.6	14.8
Lillian	BW621 * 3/90B07-AU2B	CNHR	2003	86.8	85.3	84.4	88.6	4.3	5.1	16.2	16.2
Lovitt	8405-JC3C * 2/BW152	CWRS	2002	86.8	86.5	87.2	95.3	4.1	5.0	16.2	16.3
AAC Tisdale	Somerset/BW865//Waskada	CWRS	2017	87.9	85.0	80.0	84.2	4.6	5.0	16.4	15.7
PT472	PT425/Helios//PT435	CWRS	-	84.8	83.2	86.7	93.0	4.7	5.0	16.0	16.0
Somerset	90B01-AD4D/Pasqua	CWRS	2005	86.5	86.1	90.5	98.7	4.0	4.5	17.6	17.7
SY479 VB	01S2004-2-13/Glenn	CWRS	2016	87.8	85.9	88.8	94.5	4.4	4.5	17.1	16.9

* CNHR: Canada Northern Hard Red; CWRS: Canada Western Red Spring; CPSR: Canada Prairie Spring Red. ** GPC: Grain protein content.

## Data Availability

All relevant files are included in this article and its Supplementary Materials files.

## Supplementary Materials

The following supporting information can be downloaded at: https://www.mdpi.com/article/10.3390/plants11141887/s1, Figure S1: Box plots of expected genetic gain (EGG) for grain yield, plant height, maturity, and grain protein content (GPC) plus response to selection and the correlations between the index and genetic merit. The plots were based on 2304 combinations of economic weights obtained using the Kempthorne and Nordskog Restrictive Linear Phenotypic Selection Index (RLPSI), and the best unbiased estimators computed from all environments regardless of the management systems. See Table S4 for details. Figure S2: Bar graph of expected genetic gain (EGG) for grain yield, grain protein content (GPC), plant height, and maturity plus response to selection (Rs) and the correlations between the index and genetic merit (r) based on 48 of the 2304 combinations of economic weights. For each scenario (GPC = 0 or Yield = 0), all economic weights were similar regarding the EGG for yield or GPC, but different in the other parameters. The five economic weights that gave higher positive EGG for yield or GPC with greater Rs and r, and the lowest (negative) EGG both for plant height and maturity were selected. The 5 selected economic weights are indicated with * with details shown in Table 1 and Table S4. Figure S3: Bar graph of expected genetic gain (EGG) for grain yield, plant height, and maturity plus response to selection and the correlations between the index and genetic merit. The plots were based on 2304 combinations of economic weights obtained using the Kempthorne and Nordskog Restrictive Linear Phenotypic Selection Index (RLPSI) and the best unbiased estimators computed from all environments regardless of the management systems. See Tables S3 and S6 for more results. Table S1: Summary of the spring wheat germplasm used in the present study, including the best linear unbiased estimators (BLUEs) for maturity (d), plant height (cm), grain yield (t ha^−1^), and grain protein content (%) computed from 5 conventionally and 4 organically managed environments. Table S2: Example R codes used to compute the best combinations of economic weights and the final analysis using the Smith Linear Phenotypic Selection Index (LPSI) and the Kempthorne and Nordskog Restrictive Linear Phenotypic Selection Index (RLPSI). In contrast to LPSI which did not impose a restriction, RLPSI was run by restricting the expected genetic gain either for grain yield or grain protein content to zero. Table S3: Summary of the cultivars selected based on either their high grain yield or grain protein content in each management system. Table S4: Summary of the selection parameters (expected genetic gain per trait, response to selection, and correlation between the index and the genetic merit) for a total of 2304 economic weights. The analyses were done using the Kempthorne and Nordskog Restrictive Linear Phenotypic Selection Index (RLPSI) and the best linear unbiased estimators (BLUEs) computed from all 9 environments regardless of the management systems. Table S5: Analysis of Variance (ANOVA) comparing expected genetic gain for grain yield at different economic weights for maturity and plant height (−1, −5, −10, −15), grain yield (1 to 150 at an increment of 10), and grain protein content (10 to 80 at an increment of 10). Table S6: Summary of the selection parameters (expected genetic gain per trait, response to selection, and correlation between the index and the genetic merit) based on five economic weights. The analyses were done using the Smith Linear Phenotypic Selection Index (LPSI) and the Kempthorne and Nordskog Restrictive Linear Phenotypic Selection Index (RLPSI) and the best linear unbiased estimators (BLUEs) were computed from 5 conventionally and 4 organically managed environments separately. Table S7: Summary of the phenotype data of 47 selected cultivars using five economic weights and the Smith Linear Phenotypic Selection Index (LPSI) and the Kempthorne and Nordskog Restrictive Linear Phenotypic Selection Index (RLPSI). Both the LPSI and RLPSI were computed using the best linear unbiased estimators (BLUEs) computed from 5 conventionally and 4 organically managed environments separately.
