# Supplementary material for: Identification of Spring Wheat with Superior Agronomic Performance under Contrasting Nitrogen Managements Using Linear Phenotypic Selection Indices
Source: Plants (Basel). 2022 Jul 20;11(14):1887. doi: 10.3390/plants11141887 (PMC9317689; doi:10.3390/plants11141887)
Supplement: Supplementary file 1 [file plants-11-01887-s001.zip › Supplementary Figures.pptx]

## Slide 1
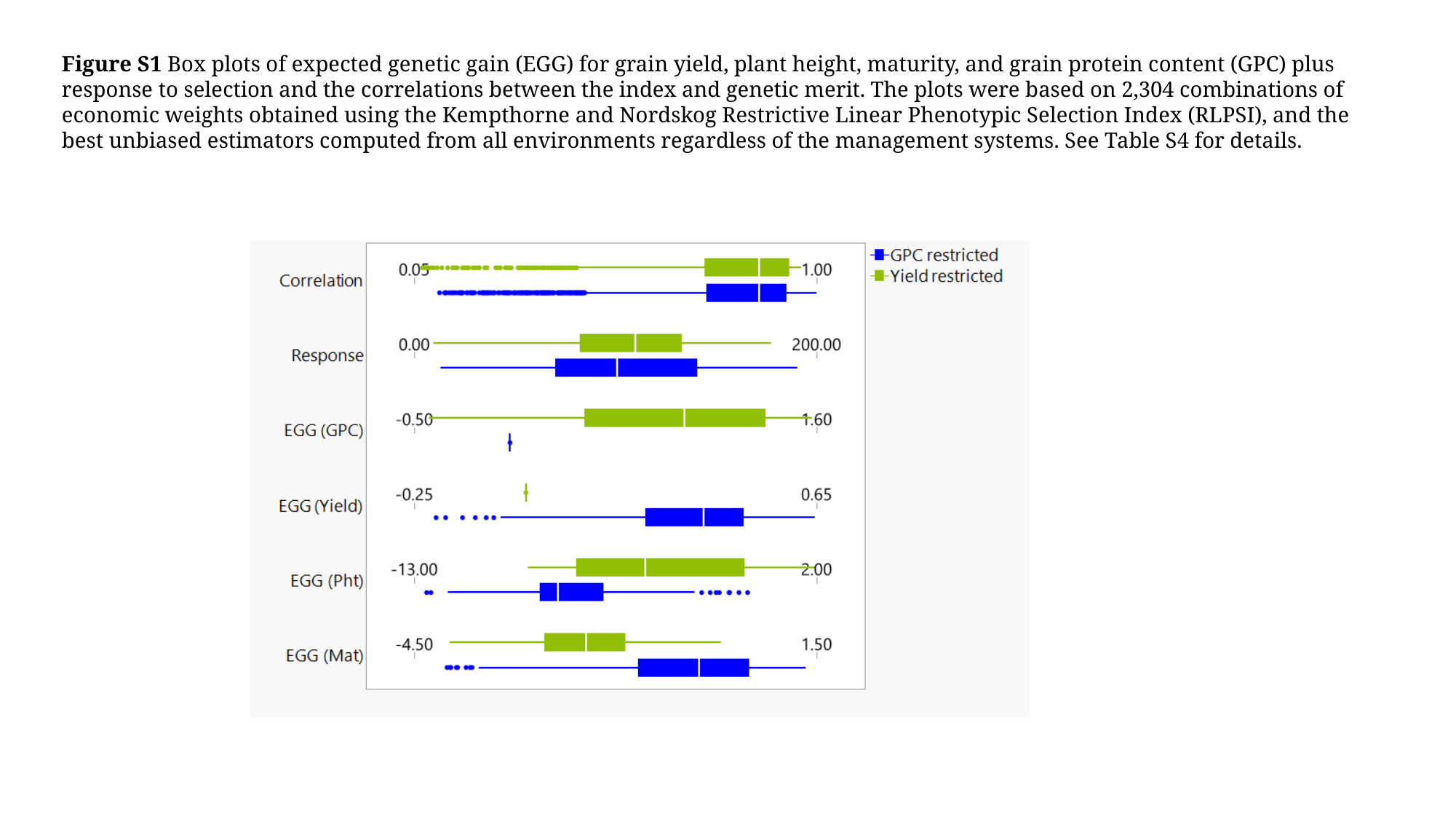

Figure S1 Box plots of expected genetic gain (EGG) for grain yield, plant height, maturity, and grain protein content (GPC) plus response to selection and the correlations between the index and genetic merit. The plots were based on 2,304 combinations of economic weights obtained using the Kempthorne and Nordskog Restrictive Linear Phenotypic Selection Index (RLPSI), and the best unbiased estimators computed from all environments regardless of the management systems. See Table S4 for details.

## Slide 2
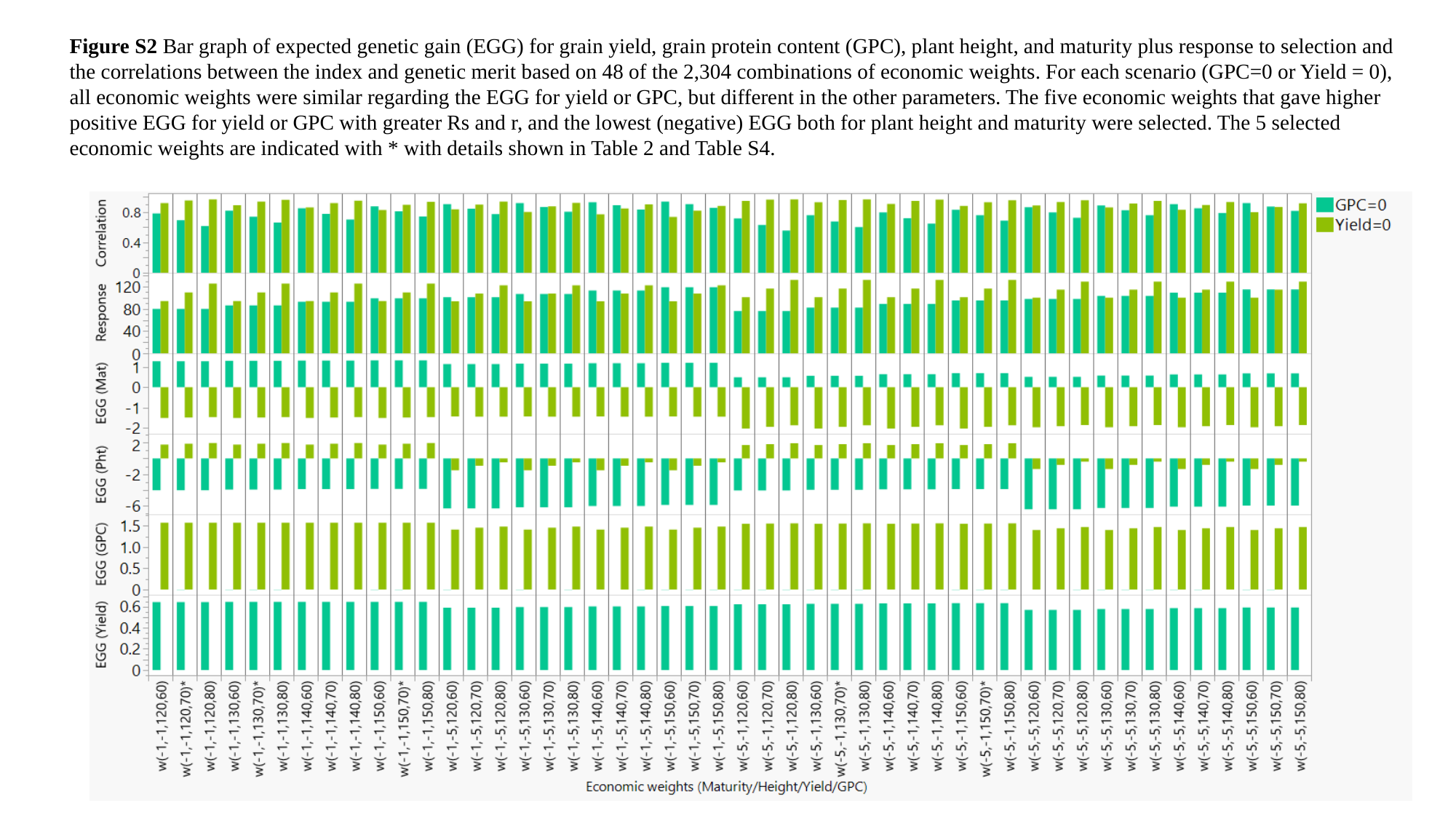

Figure S2 Bar graph of expected genetic gain (EGG) for grain yield, grain protein content (GPC), plant height, and maturity plus response to selection and the correlations between the index and genetic merit based on 48 of the 2,304 combinations of economic weights. For each scenario (GPC=0 or Yield = 0), all economic weights were similar regarding the EGG for yield or GPC, but different in the other parameters. The five economic weights that gave higher positive EGG for yield or GPC with greater Rs and r, and the lowest (negative) EGG both for plant height and maturity were selected. The 5 selected economic weights are indicated with * with details shown in Table 2 and Table S4.

## Slide 3
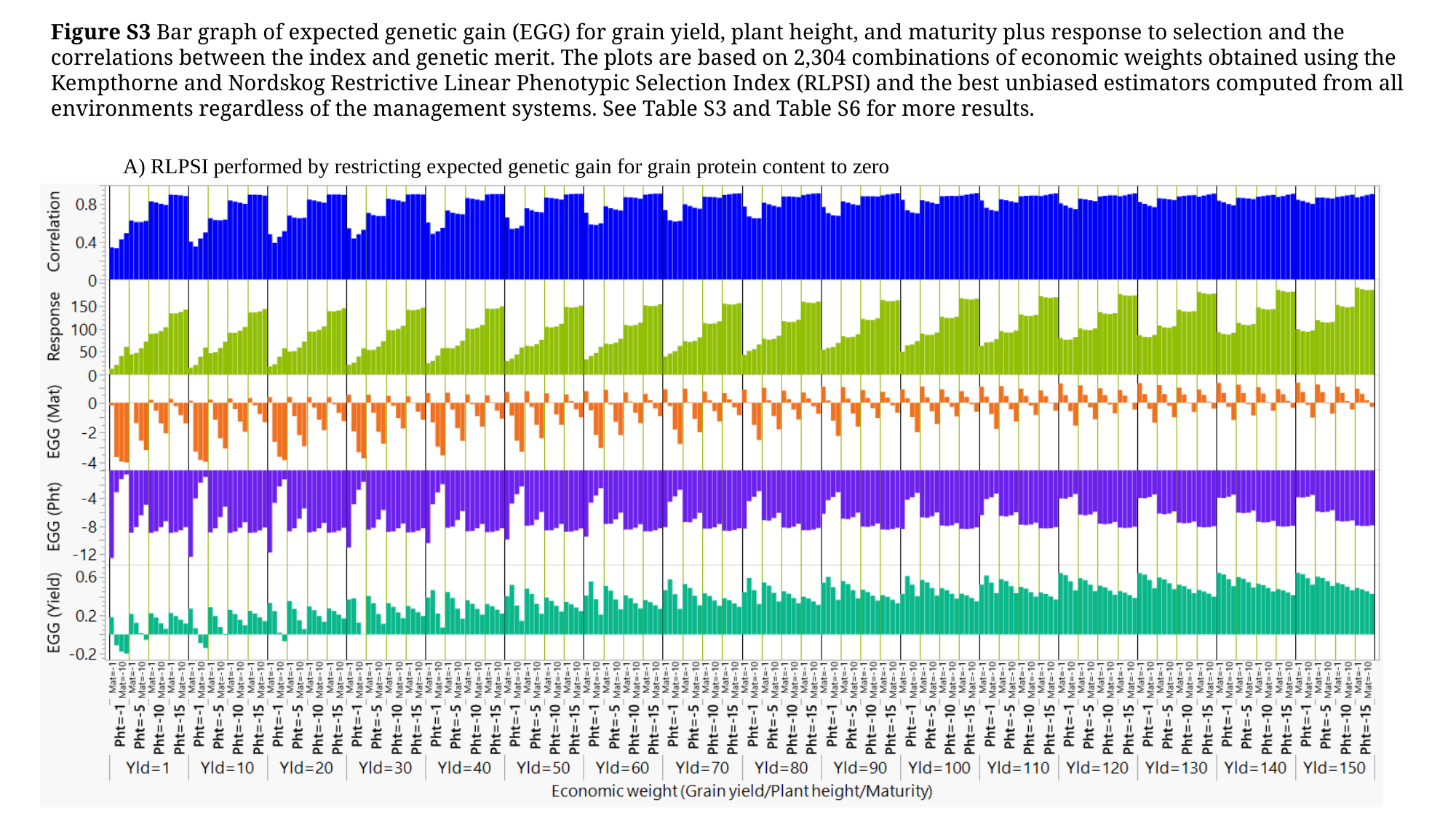

Figure S3 Bar graph of expected genetic gain (EGG) for grain yield, plant height, and maturity plus response to selection and the correlations between the index and genetic merit. The plots are based on 2,304 combinations of economic weights obtained using the Kempthorne and Nordskog Restrictive Linear Phenotypic Selection Index (RLPSI) and the best unbiased estimators computed from all environments regardless of the management systems. See Table S3 and Table S6 for more results.
A) RLPSI performed by restricting expected genetic gain for grain protein content to zero

## Slide 4
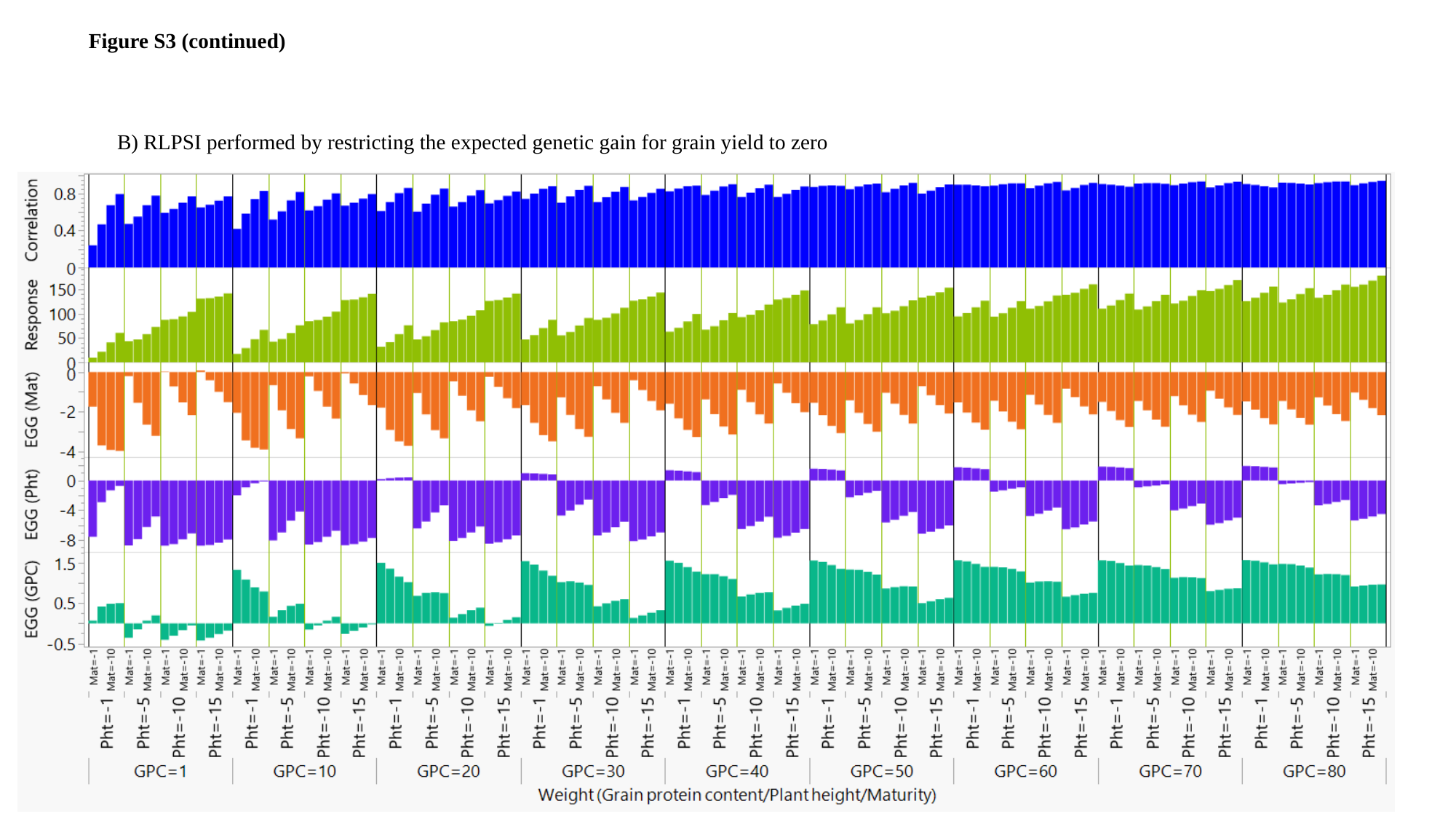

Figure S3 (continued)
B) RLPSI performed by restricting the expected genetic gain for grain yield to zero
