# Supplementary material for: Identification of Spring Wheat with Superior Agronomic Performance under Contrasting Nitrogen Managements Using Linear Phenotypic Selection Indices
Source: Plants (Basel). 2022 Jul 20;11(14):1887. doi: 10.3390/plants11141887 (PMC9317689; doi:10.3390/plants11141887)
Supplement: Supplementary file 1 [file plants-11-01887-s001.zip › Table S5 29042022.pdf]

Table S5 Analysis of Variance (ANOVA) comparing expected genetic gain for grain yield at different economic weights for maturity and plant height (-1, -5, -10, -15), grain yield (1 to 150 at an increment of 10), and grain protein content (10 to 80 at an increment of 10).

A) Comparison of economic weight based on Kempthorne and Nordskog Restrictive Linear Phenotypic Selection Index (RLPSI) by imposing restrictions to grain protein content (GPC)

Y (Response) = Expected genetic gain for grain yield
X (Factor): Grain yield economic weight

Analysis of Variance

Source	DF	Sum of Squares	Mean Square	F Ratio	Prob > F
Weight (Yield)	15	44.487936	2.96586	341.6285	<.0001*
Error	2288	19.863369	0.00868		
Total	2303	64.351305			

Means Comparisons using Tukey-Kramer HSD

Level													Mean yield
Yld=150	A												0.53321794
Yld=140	A												0.52343145
Yld=130	A	B											0.51240962
Yld=120	A	B											0.49994566
Yld=110		B	C										0.47828355
Yld=100			C										0.45608552
Yld=90			C	D									0.44511034
Yld=80				D	E								0.41773606
Yld=70					E	F							0.39466950
Yld=60						F	G						0.36311640
Yld=50							G						0.33006452
Yld=40								H					0.29163953
Yld=30									I				0.24690532
Yld=20										J			0.19495025
Yld=10											K		0.13567574
Yld=1												L	0.07764909

Levels not connected by same letter are significantly different.

Y (Response) = Expected genetic gain for grain yield
X (Factor): Maturity economic weight

Analysis of Variance

**Source**	**DF**	**Sum of Squares**	**Mean Square**	**F Ratio**	**Prob > F**
Weight (Mat)	3	9.285581	3.09519	129.2809	<.0001*
Error	2300	55.065724	0.02394		
Total	2303	64.351305			

Means Comparisons using Tukey-Kramer HSD

Level				Mean yield
Mat=-1	A			0.43630416
Mat=-5	A			0.41752657
Mat=-10		B		0.34560690
Mat=-15			C	0.27578500

Levels not connected by same letter are significantly different.

Y (Response) = Expected genetic gain for grain yield
X (Factor): Plant height economic weight

Analysis of Variance

Source	DF	Sum of Squares	Mean Square	F Ratio	Prob > F
Weight (Height)	3	1.208045	0.402682	14.6677	<.0001*
Error	2300	63.143260	0.027454		
Total	2303	64.351305			

Means Comparisons using Tukey-Kramer HSD

Level				Mean yield
Pht=-5	A			0.39794774
Pht=-1	A	B		0.37909706
Pht=-10		B		0.36259126
Pht=-15			C	0.33558657

Levels not connected by same letter are significantly different.

Y (Response) = Expected genetic gain for grain yield
X (Factor): GPC economic weight

Analysis of Variance

Source	DF	Sum of Squares	Mean Square	F Ratio	Prob > F
Weight (GPC)	8	0.001487	0.000186	0.0066	1.0000
Error	2295	64.349818	0.028039		
Total	2303	64.351305			

Means Comparisons using Tukey-Kramer HSD

Level		Mean yield
GPC=60	A	0.37022916
GPC=30	A	0.37022314
GPC=70	A	0.36853279
GPC=20	A	0.36852193
GPC=40	A	0.36852193
GPC=80	A	0.36852193
GPC=10	A	0.36851490
GPC=01	A	0.36851490
GPC=50	A	0.36767023

Levels not connected by same letter are significantly different.

Y (Response) = Expected genetic gain for maturity
X (Factor): Maturity economic weight

Analysis of Variance

Source	DF	Sum of Squares	Mean Square	F Ratio	Prob > F
Weight (Mat)	3	1871.5172	623.839	855.1635	<.0001*
Error	2300	1677.8428	0.729		
C. Total	2303	3549.3600			

Means Comparisons using Tukey-Kramer HSD

Level					Mean days
Mat=-01	A				0.747199
Mat=-05		B			-0.106851
Mat=-10			C		-0.973077
Mat=-15				D	-1.646905

Levels not connected by same letter are significantly different.

Y (Response) = Expected genetic gain for plant height
X (Factor): Plant height economic weight

Analysis of Variance

Source	DF	Sum of Squares	Mean Square	F Ratio	Prob > F
Weight (Height)	3	5139.727	1713.24	767.0369	<.0001*
Error	2300	5137.247	2.23		
C. Total	2303	10276.974			

Means Comparisons using Tukey-Kramer HSD

Level					Mean height
Pht=-01	A				-4.547960
Pht=-05		B			-6.754212
Pht=-10			C		-7.957483
Pht=-15				D	-8.400811

Levels not connected by same letter are significantly different.

B) Comparison of economic weight based on Kempthorne and Nordskog Restrictive Linear Phenotypic Selection Index (RLPSI) by imposing restrictions to grain yield

Y (Response) = Expected genetic gain for grain protein content (GPC)
X (Factor): Grain protein content economic weights

Analysis of Variance

Source	DF	Sum of Squares	Mean Square	F Ratio	Prob > F
Weight (GPC)	8	422.99193	52.8740	377.1850	<.0001*
Error	2295	321.71432	0.1402		
Total	2303	744.70625			

Means Comparisons using Tukey-Kramer HSD

Level									Mean GPC
GPC=80	A								1.287517
GPC=70	A	B							1.229453
GPC=60		B	C						1.156006
GPC=50			C						1.062484
GPC=40				D					0.942566
GPC=30					E				0.787448
GPC=20						F			0.584506
GPC=10							G		0.313688
GPC=1								H	-0.055829

Levels not connected by same letter are significantly different.

Y (Response) = Expected genetic gain for grain protein content
X (Factor): Maturity economic weights

Analysis of Variance

Source	DF	Sum of Squares	Mean Square	F Ratio	Prob > F
Weight (Mat)	3	0.52160	0.173866	0.5374	0.6567
Error	2300	744.18465	0.323559		
C. Total	2303	744.70625			

Means Comparisons using Tukey-Kramer HSD

Level		Mean GPC
Mat=-10	A	0.82475439
Mat=-15	A	0.81866982
Mat=-5	A	0.81819527

Level		Mean GPC
Mat=-1	A	0.78630935

Levels not connected by same letter are significantly different.

Y (Response) = Expected genetic gain for grain protein content
X (Factor): Plant height economic weights

Analysis of Variance

Source	DF	Sum of Squares	Mean Square	F Ratio	Prob > F
Weight (Height)	3	274.28229	91.4274	447.0076	<.0001*
Error	2300	470.42397	0.2045		
C. Total	2303	744.70625			

Means Comparisons using Tukey-Kramer HSD

Level					Mean GPC
Pht=-1	A				1.2801727
Pht=-5		B			0.9738981
Pht=-10			C		0.6239099
Pht=-15				D	0.3699480

Levels not connected by same letter are significantly different.

Y (Response) = Expected genetic gain for grain protein content
X (Factor): Grain yield economic weights

Analysis of Variance

Source	DF	Sum of Squares	Mean Square	F Ratio	Prob > F
Weight (Yield)	15	1.2247e-28	8.16e-30	0.0000	1.0000
Error	2288	744.70625	0.325484		
C. Total	2303	744.70625			

Means Comparisons using Tukey-Kramer HSD

Level		Mean GPC
Yld=1	A	0.81198221
Yld=20	A	0.81198221
Yld=40	A	0.81198221
Yld=80	A	0.81198221
Yld=10	A	0.81198221
Yld=50	A	0.81198221
Yld=30	A	0.81198221

Level		Mean GPC
Yld=100	A	0.81198221
Yld=140	A	0.81198221
Yld=60	A	0.81198221
Yld=70	A	0.81198221
Yld=90	A	0.81198221
Yld=110	A	0.81198221
Yld=150	A	0.81198221
Yld=120	A	0.81198221
Yld=130	A	0.81198221

Levels not connected by same letter are significantly different.

Y (Response) = Expected genetic gain for maturity
X (Factor): maturity economic weights

Analysis of Variance

Source	DF	Sum of Squares	Mean Square	F Ratio	Prob > F
Weight (Mat)	3	909.6436	303.215	729.2272	<.0001*
Error	2300	956.3459	0.416		
Total	2303	1865.9895			

Means Comparisons using Tukey-Kramer HSD

Level					Mean days
Mat=-1	A				-1.025559
Mat=-5		B			-1.717708
Mat=-10			C		-2.293482
Mat=-15				D	-2.694246

Levels not connected by same letter are significantly different.

Y (Response) = Expected genetic gain for plant height
X (Factor): Plant height economic weights

Analysis of Variance

Source	DF	Sum of Squares	Mean Square	F Ratio	Prob > F
Weight (Height)	3	19079.516	6359.84	1815.211	<.0001*
Error	2300	8058.364	3.50		
Total	2303	27137.880			

Means Comparisons using Tukey-Kramer HSD

Level					Mean height
Pht=-1	A				0.583729
Pht=-5		B			-3.149667
Pht=-10			C		-5.679718
Pht=-15				D	-6.954490

Levels not connected by same letter are significantly different.
